# Synovial Sarcoma in the Right Thigh of a 25-Year-Old Male: A Case Report

**DOI:** 10.7759/cureus.62526

**Published:** 2024-06-17

**Authors:** Chahat Singh, Pankaj Gharde, Rakshit Shah, Tushar Nagtode, Bhagyesh Sapkale

**Affiliations:** 1 General Surgery, Jawaharlal Nehru Medical College, Datta Meghe Institute of Higher Education and Research, Wardha, IND; 2 Surgical Oncology, Care Super Speciality Hospital, Vadodara, IND; 3 Medicine, Jawaharlal Nehru Medical College, Datta Meghe Institute of Higher Education and Research, Wardha, IND

**Keywords:** bone and soft tissue sarcoma, rotation flap, aggressive sarcoma, synovial sarcoma, sarcoma soft tissue

## Abstract

In this article, we describe the diagnosis and surgical treatment of synovial sarcoma within the right thigh of a 25-year-old male. Our article aims to demonstrate successful treatment through wide local excision and rotation flap reconstruction, emphasizing the noteworthiness of comprehensive surgical planning and postoperative care. Postoperative care guaranteed satisfactory healing and appendage functionality, with a follow-up plan to monitor for recurrence. This case underscores the need for individualized treatment approaches and highlights the significance of multidisciplinary care for overseeing aggressive tumors like synovial sarcoma. The findings contribute to the ongoing refinement of surgical procedures and postoperative protocols to improve patient results.

## Introduction

A lipoma is a benign, slowly growing, circular, or oval-shaped lump of fatty tissue that commonly feels smooth to the touch and moves instantly when touched [1]. It is most commonly experienced on the back, trunk, arms, shoulders, and neck [1]. A sarcoma could be a cancer that starts inside the body's connective tissues, consolidating cartilage, blood vessels, muscles, bones, and fat [2]. Sarcomas are perilous tumors that can spread to other body regions and attack enveloping tissues, in contrast to lipomas, which are benign and made of fat cells [3]. Soft tissue and bone sarcomas are the two basic sarcomas [4,5]. Malignant tumors are known as bone sarcomas, which regularly influence the long bones of the arms and legs, and incorporate osteosarcoma (cancer starting in bone-forming cells), Ewing's sarcoma (cancer emerging within the bones or delicate tissue encompassing the bones), and chondrosarcoma (cancer beginning in cartilage cells) [1,3,4].

Soft tissue sarcomas (STS) can be observed in any soft tissue of the body and incorporate liposarcoma (emerging from fat tissue), leiomyosarcoma (starting from smooth muscle tissue), and angiosarcoma (developing from blood vessels) among others [5]. STS such as synovial sarcoma are not limited to synovial tissues; they can also develop in the vicinity of bursae, tendons, and joint capsules [6]. It frequently affects the soft tissues that surround joints as well as the intermuscular planes [6]. Unbelievably, considering its name, synovial sarcoma is not caused by synovial cells but rather by primitive mesenchymal cells that can develop into spindle cells and epithelial-like components [3,5,6]. For synovial sarcoma, wide local excision surgery is a typical treatment strategy [6]. One surgical procedure used in reconstructive surgery to fix soft tissue or skin deformities is known as the rotation flap [6]. STS predominantly occur in the extremities and are more common than bone sarcomas, with an incidence rate of approximately five per 100,000 people annually and an STS to bone sarcoma ratio of about 3:1 [1,4,5]. Although less common, retroperitoneal sarcomas represent a significant challenge due to their anatomical location [7]. Achieving a complete resection with negative margins (R0 resection), where no cancer cells are seen at the resection margin, is the cornerstone of effective sarcoma treatment [7].

## Case presentation

**Figure 1:**
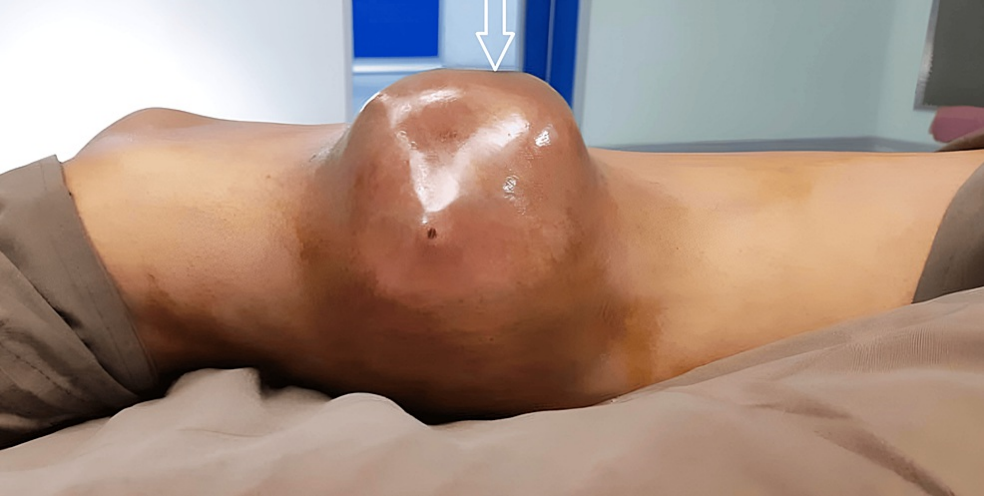
Swelling on the right thigh

A 25-year-old male presented to the outpatient department of Acharya Vinoba Bhave Rural Hospital (AVBRH) with a swelling on the right thigh that had been progressively increasing for the past year, as shown in Figure 1.

**Figure 2:**
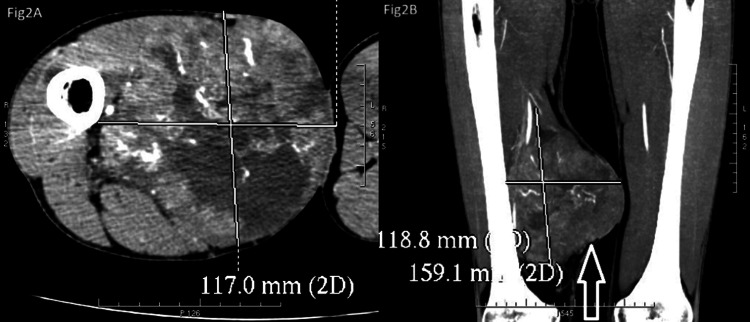
Computed tomography (CT) scans of the right lower limb (A) Anteroposterior section view of the right lower limb depicting the tumor. (B) Coronal section view of the right lower limb with the white arrow indicating the synovial sarcoma.

**Figure 3:**
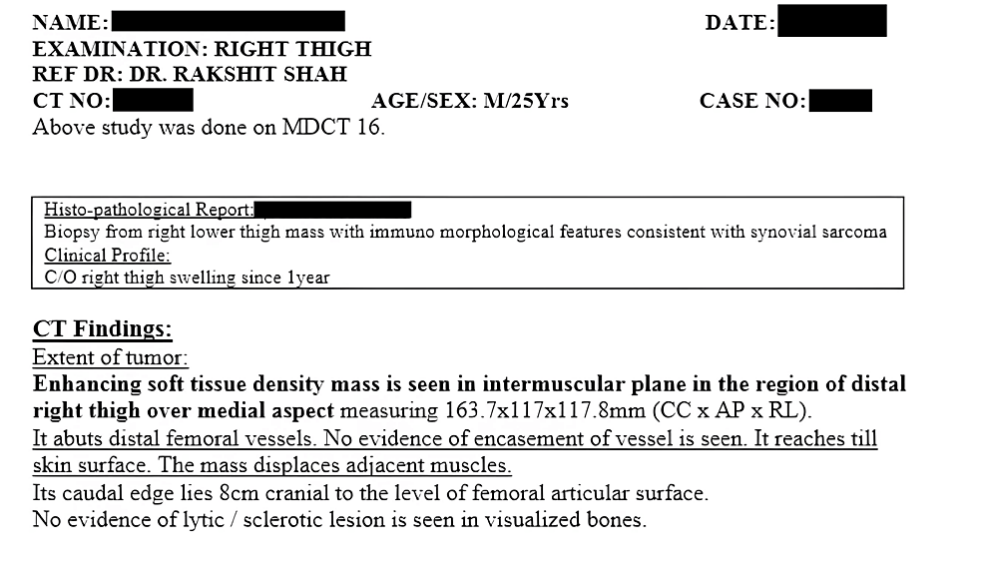
Histopathology report of the patient The histopathology report of the patient confirming synovial sarcoma is shown. Demographic and patient-identifying details are hidden to address the privacy concerns of the given case.

On examination, the mass was located in the distal region of the right thigh over the medial aspect, measuring 163.7 mm craniocaudal (CC) x 117 mm anteroposterior (AP) x 117.8 mm right-left (RL). The soft tissue density mass was situated in the intermuscular plane and abutted the distal femoral vessels without evidence of vascular encasement. It reached the skin surface and displaced adjacent muscles without restricting their movements. A computed tomography (CT) scan revealed the caudal edge of the tumor, lying 8 cm cranial to the level of the femoral articular surface, with no evidence of lytic lesions in the visualized bones. Subsequent histopathological analysis confirmed the diagnosis of synovial sarcoma. CT scans of the right lower limb of the patient showing synovial sarcoma are presented in Figure 2. The histopathology report of the patient is presented in Figure 3.

**Figure 4:**
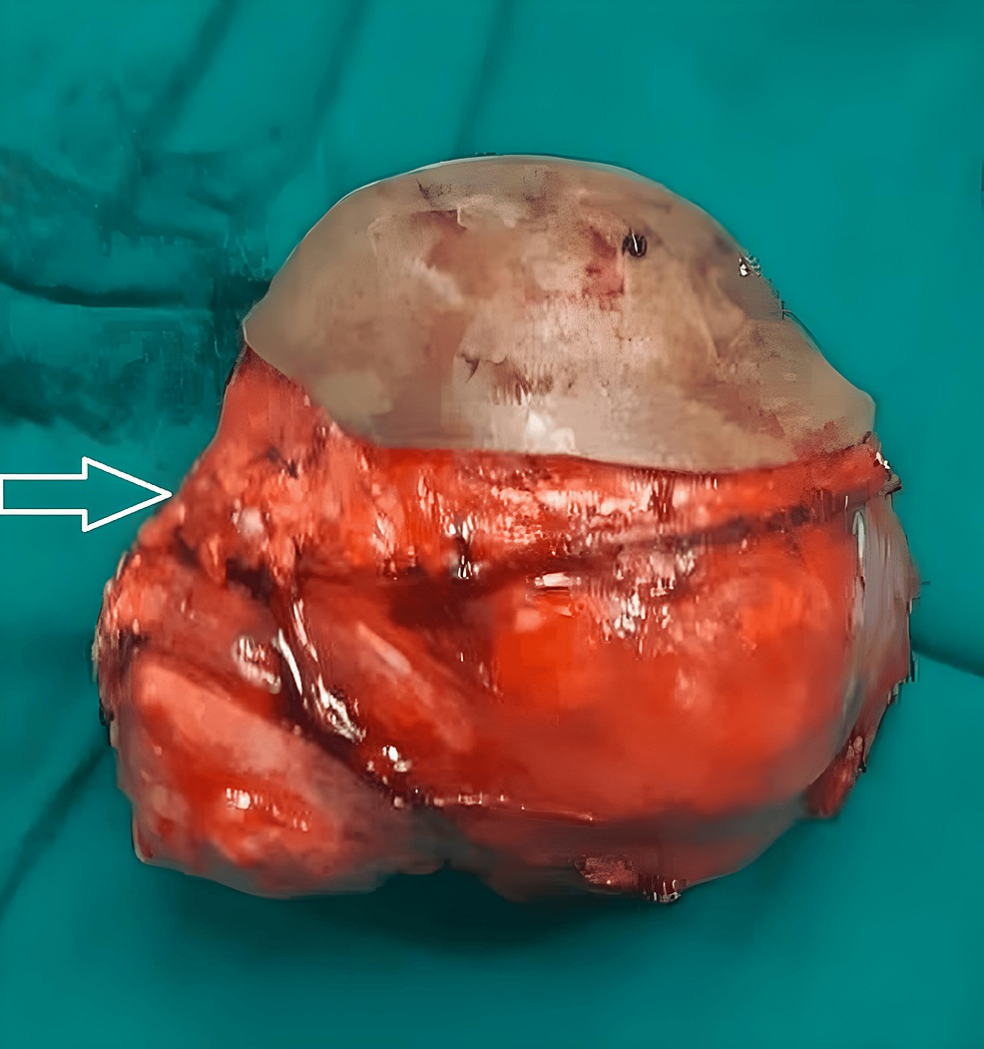
The excised sarcoma specimen

The patient underwent a wide local excision to remove the sarcoma with clear margins. Under general anesthesia, a longitudinal incision was made over the medial aspect of the right thigh. Careful dissection through the subcutaneous tissue allowed for visualization of the tumor within the intermuscular plane. The tumor was excised with a wide margin of healthy tissue, ensuring complete removal while preserving the distal femoral vessels. Hemostasis was achieved using electrocautery, and the wound was closed in layers with the deep tissues sutured using absorbable sutures and the skin closed with non-absorbable sutures. A sterile dressing was applied to the incision site. The excised sarcoma is shown in Figure 4.

To cover the defect made by the tumor excision, a rotation flap was outlined and executed. The flap was carefully arranged in the lateral aspect of the right thigh to guarantee adequate measurements and a proper blood supply. An incision was made around the outlined flap, and it was dissected from the underlying tissues while protecting its vascular pedicle. The vascular pedicle was based on the perforating branches of the profunda femoris artery, ensuring adequate blood supply to the rotated flap. The flap was at that point rotated into the imperfection and secured with hindered sutures. The given site was closed with essential sutures, and in a few zones, a skin graft was connected to guarantee total coverage. The surgical site was then dressed with a sterile dressing.

Postoperative care included observing the flap for satisfactory perfusion and healing, pain administration, antibiotics, and wound care. The surgery effectively evacuated the synovial sarcoma with clear edges, giving satisfactory coverage of the imperfection, and protected appendage function without limiting movement. The patient was prompted to customary follow-up for two years to screen for recurrence and was alluded to physical treatment to guarantee full utilitarian recovery. Proceeded imaging and clinical assessments were arranged as a part of this long-term follow-up. The timeline of events in the given case report is described in Table 1.

**Table 1:** Timeline of events in the given case Table created by Chahat Singh and Bhagyesh Sapkale

Event	Day	Description
Initial presentation	1	A 25-year-old male presented at Acharya Vinoba Bhave Rural Hospital (AVBRH) with a growing swelling on the right thigh's medial aspect (for the past one year)
Examination	1 (Shortly after presentation)	Mass on the distal, medial aspect of the right thigh (size: 163.7 mm craniocaudal (CC) x 117 mm anteroposterior (AP) x 117.8 mm right-left (RL)): soft tissue density, intermuscular plane; abuts distal femoral vessels (no encasement); reaches skin surface, displaces muscles (no movement restriction)
CT scan	1-3 (After examination)	Revealed tumor 8 cm cranial to femoral articular surface, no bone lesions
Diagnosis	1-3 (Following CT scan)	Synovial sarcoma diagnosed based on findings
Surgery	5 (Preparation and scheduling)	Wide local excision performed under general anesthesia: longitudinal incision on medial right thigh; dissection through the subcutaneous tissue to visualize the tumor; excision of the tumor with clear margins, preserving distal femoral vessels. Hemostasis achieved with electrocautery: wound closed in layers (absorbable sutures for deep tissues, non-absorbable for skin); sterile dressing applied
Flap reconstruction	Same day as surgery (Following excision)	The rotation flap designed and executed from the lateral aspect of the right thigh to cover the excision defect. Flap design ensures sufficient size and blood supply based on perforating branches of the profunda femoris artery. Incision made, flap dissected while preserving blood supply. Flap rotated and secured to defect with sutures. Donor site closed with primary sutures; skin graft used in some areas. Surgical site dressed with a sterile dressing
Postoperative care	Began the day after surgery	Monitor flap perfusion and healing; pain management; antibiotics; wound care
Hospital discharge	5 days post-surgery	The patient was discharged with continued follow-up care
Follow-up plan (Regular intervals)	Began after discharge	Regular follow-up for two years to monitor for recurrence. Referral to physical therapy for full functional recovery. Continued imaging and clinical assessments

## Discussion

Our case of the 25-year-old male with synovial sarcoma of the right thigh offers a few similitudes and differences with the case detailed by Serinelli et al. of a 69-year-old Caucasian female with a dorsal ulnar-sided left hand wrist mass [8]. Both cases incorporate the diagnosis and surgical treatment of synovial sarcoma, an exceptional and aggressive soft tissue tumor. In both events, the patients experienced wide surgical excision of the mass to ensure the whole evacuation of the tumor. Besides, the case of the 69-year-old female utilized advanced demonstrative strategies, such as immunohistochemical staining and fluorescence in situ hybridization (FISH) for SS18 (SYT) gene improvement, to certify the diagnosis of synovial sarcoma [8].

In our case of the 25-year-old male, the patient had a giant tumor inside the right thigh, compared to the much smaller 1.0 x 0.5 x 0.5 cm mass inside the female patient's left hand wrist [8]. The 25-year-old male patient required a rotation flap to cover the flaw made by the tumor extraction, whereas the female patient's treatment included en-bloc resection of the distal ulna [8]. Also, our male patient did not report a past injury or side effects history. To differentiate, the female patient had a decade-long history of continuously developing and dynamically painful mass. In addition, the 69-year-old female patient got adjuvant radiotherapy post-surgery and had standard follow-ups over 5.5 years without recurrence [8]. These cases highlight the changeability in presentation, measurement, and complexity of synovial sarcoma cases, as well as the custom-made surgical and postoperative approaches required for diverse anatomical areas and patient histories.

In the case of a 30-year-old woman reported by Namazi et al., the patient had a two-year history of an erythematous, slow-growing papule on the left thigh that enlarged during pregnancy, ultimately becoming a solid, ulcerated subcutaneous nodule with prominent surface vessels [9]. The histopathological examination revealed a rather well-circumscribed dermal neoplasm extending into subcutaneous tissue with high mitotic activity and immunohistochemical positivity for vimentin, CD99, and Bcl-2, but not for markers like CD45, HMB45, and S-100 [9]. The case required a second surgery due to the involved tumor margins. Conversely, our case of the 25-year-old male presented with a significantly larger synovial sarcoma on his right thigh, measuring 163.7 mm CC x 117 mm AP x 117.8 mm RL, and underwent a wide local excision followed by a rotation flap to cover the defect. Both cases highlight the importance of thorough histopathological and immunohistochemical analysis for accurate diagnosis, as well as the need for surgical intervention to achieve clear margins, though the extent of the surgical procedures differed due to the size and clinical presentation of the tumors [9].

A case reported by Ghimire et al. details a 13-year-old female who displayed an uncovered synthetic femoral vessel graft, a complication from numerous surgical intercessions for synovial sarcoma within the right thigh [10]. At first, she presented with swelling and pain, analyzed through magnetic resonance imaging (MRI) and immunohistochemistry. She underwent wide local excision (WLE) and femoral vessel reconstruction with a manufactured graft but experienced postoperative complications, including synthetic graft thrombosis and graft exposure, requiring encouraged surgical interventions [10]. Closure of the exposed graft was accomplished by employing a vertical rectus abdominis myocutaneous (VRAM) flap, and a thrombectomy of the synthetic artery graft was performed [10]. Her postoperative recuperation was palatable and regular follow-ups showed no recurrence or metastasis. In comparison to our case of synovial sarcoma, both these cases highlight the complexity of managing synovial sarcoma with vascular inclusion and emphasize the significance of multidisciplinary care. Finishing the clear edges was important in both cases, with the usage of WLE [10].

Another case described by Akhanoba et al. involving a 28-year-old woman with a complex medical history presents several similarities and differences when compared to our case of synovial sarcoma [11]. Both individuals presented with progressively enlarging masses in the thigh region, which subsequent imaging and histological analysis confirmed as synovial sarcoma. In this case, the female patient was already managing human immunodeficiency virus (HIV) and had a history of poor antiretroviral therapy compliance so she faced a more intricate clinical trajectory. Discovery of her thigh mass during pregnancy added layers of complexity to her treatment [11]. Ultrasound and MRI scans revealed a large heterogeneous soft tissue mass encasing femoral vessels and infiltrating adjacent muscles. Despite initially detectable HIV viral load, an intensified antiretroviral regimen reduced it to undetectable levels pre-surgery [11]. Due to the tumor’s aggressiveness and vessel encasement, she underwent hip disarticulation surgery at 30 weeks of pregnancy following multidisciplinary team deliberation. Her postoperative course was complicated by sepsis from an infected leg stump, necessitating multiple wound debridement and prolonged intravenous antibiotics [11]. She conveyed her child through a crisis cesarean segment at 34 weeks. We too utilized a multidisciplinary approach including orthopedic specialists, oncologists, radiologists, and physical specialists. These cases emphasize the significance of specialized expert centers, the requirement of personalized, multidisciplinary approaches in synovial sarcoma administration, legitimate treatment to individual understanding components, and comorbidities to optimize outcomes.

## Conclusions

This case reports the compelling organization of a synovial sarcoma in a 25-year-old male through wide local excision and rotation flap reconstruction, underscoring the noteworthiness of comprehensive surgical planning and postoperative care. Future inquiries ought to focus on refining surgical techniques and postoperative conventions to improve recovery and minimize recurrence. Additionally, the case emphasizes the requirement for individualized treatment approaches, particularly for unprecedented and aggressive tumors like synovial sarcoma. Continued progressions in diagnostic imaging and molecular pathology will be noteworthy in making strides in early discovery and custom-made therapeutic methods for such complex cases. Achieving R0 resection, or complete removal of the tumor with negative surgical margins, is crucial in managing cancers like synovial sarcoma. This ensures that all cancerous cells are removed, minimizing the risk of recurrence and improving long-term outcomes for patients. Specialized centers are critical in this process by providing access to experienced healthcare professionals, various diagnostic tools, and advanced treatment modalities. These centers are equipped to handle complex cases like synovial sarcoma, requiring a multidisciplinary approach involving surgeons, oncologists, radiologists, and pathologists working together to plan and execute the most effective treatment strategy.

## References

[1] Johnson CN, Ha AS, Chen E, Davidson D (2018). Lipomatous soft-tissue tumors. J Am Acad Orthop Surg.

[2] Rassy E, Abou-Jaoude R, Boussios S, Assi T, Kattan J, Khaled H, Pavlidis N (2022). Sarcoma of unknown primary: myth or reality?. J Egypt Natl Canc Inst.

[3] Miwa S, Yamamoto N, Tsuchiya H (2023). Sarcoma: molecular pathology, diagnostics, and therapeutics. Int J Mol Sci.

[4] Ferguson JL, Turner SP (2018). Bone cancer: diagnosis and treatment principles. Am Fam Physician.

[5] Gilbert NF, Cannon CP, Lin PP, Lewis VO (2009). Soft-tissue sarcoma. J Am Acad Orthop Surg.

[6] Gazendam AM, Popovic S, Munir S, Parasu N, Wilson D, Ghert M (2021). Synovial sarcoma: a clinical review. Curr Oncol.

[7] Verras GI, Mulita F, Bouchagier K (2022). Mid-term outcomes in the treatment of retroperitoneal sarcomas: a 12-year single-institution experience. Med Glas (Zenica).

[8] Serinelli S, Gitto L, Zaccarini DJ (2021). Synovial sarcoma of the hand-wrist: a case report and review of the literature. J Med Case Rep.

[9] Namazi N, Ghassemipour M, Rakhshan A, Abbasi A (2016). Primary cutaneous synovial sarcoma: an extremely rare report of superficial synovial sarcoma. Indian J Dermatol.

[10] Ghimire S, Pokhrel P, Thapa S (2023). Limb conservation surgery in biphasic synovial sarcoma of thigh with vascular involvement: a race against time. Int J Surg Case Rep.

[11] Akhanoba F, Zill-E-Huma R, Pollock R, Edwards SJ (2019). Synovial sarcoma in an HIV-positive pregnant woman and review of literature. BMJ Case Rep.

